# Topical oxybutynin deodorant for axillary hyperhidrosis: a topic or a systemic effect? Rationale and design of the phase II today trial

**DOI:** 10.1590/1677-5449.202400982

**Published:** 2025-02-21

**Authors:** Samantha Neves, Giuliano Giova Volpiani, Alexia Paganotti, Felipe Menegueti, Ricardo Sanchez Boix, Roberto Augusto Caffaro, Vanessa Prado dos Santos, Eduardo Ramacciotti

**Affiliations:** 1 Irmandade Santa Casa de Misericórdia de São Paulo, São Paulo, SP, Brasil.; 2 Universidade Federal da Bahia, Faculdade de Medicina da Bahia, Salvador, BA, Brasil.; 3 Science Valley Research Institute, São Paulo, SP, Brasil.; 4 Loyola University Medical Center, Hemostasis & Thrombosis Research Laboratories, Maywood, IL, EUA.

**Keywords:** hyperhidrosis, oxybutynin, anticholinergic, axillary hyperhidrosis

## Abstract

Anticholinergics have been shown to enhance quality of life and reduce sweat in patients with hyperhidrosis. However, it remains unclear whether topical application specifically exerts local or systemic effects. This study’s primary aim is to assess topical oxybutynin’s impact on axillary hyperhidrosis. Twenty patients will be randomized into three groups. Group A will receive 2.5 mg of oral oxybutynin from day 1 to day 35 (on a variable frequency regimen). Group B will be administered a topical placebo for 35 days and Group C will receive a 10% oxybutynin topical spray, to be used twice daily for 35 days. The primary efficacy outcome will be the evaluation of the effectiveness of topical oxybutynin spray in treating hyperhidrosis. The TODAY trial will generate high-quality evidence on the effects of topical oxybutynin, assessing whether its impact is local or systemic in patients with axillary hyperhidrosis.

## INTRODUCTION

Hyperhidrosis (HH) is a condition characterized by excessive sweating, exceeding the level necessary for regulation of body temperature.^1^ It affects approximately 15.3 million individuals in North America.^2^ The etiology of primary HH remains poorly defined, but it is believed that it involves hyperactivity of the sympathetic nervous system caused by genetic predispositions.^3^ Hyperhidrosis has a significant impact on quality of life (QoL) and is associated with similar social and professional stigma to chronic conditions such as severe psoriasis. Individuals with HH also have higher rates of depression and anxiety.^4^ Primary HH, also known as idiopathic HH, accounts for around 90% of HH cases and the armpits are the most affected area.^3^ This form of HH generally starts in adolescence and tends to worsen over the years.^5-7^

Current treatment options for HH include video-assisted thoracoscopic sympathectomy (VATS), botulinum toxin injections, a range of topical treatments, and oral anticholinergics, but efficacy is variable and they can cause significant side effects.^8,9^ A long-term study involving 1,685 cases of primary HH treated with oral oxybutynin demonstrated improved quality of life and reduced HH severity, as measured by the hyperhidrosis disease severity scale (HDSS), although 24.9% of the patients experienced intense dry mouth.^10^ There are a limited number of reports of use of topical anticholinergics and of topical glycopyrrolate in the form of pads and creams, and of oxybutynin in gel form, which demonstrated efficacy.^11-14^

## BACKGROUND AND STUDY OBJECTIVES

Topical oxybutynin spray is easier to use, but has not previously been tested. The benefit of using an oxybutynin 10% spray for axillary HH would be expected to accrue from its limited systemic effects, which could reduce the sympathomimetic side effects observed with oral anticholinergic and so potentially improve adherence to treatment, improving both QoL questionnaire scores^15^ and HDSS scores.^16^

This phase II trial will assess the efficacy of a topical oxybutynin 10% spray applied twice a day, in comparison to a placebo and to oral oxybutynin in patients with axillary HH.

## METHODS

### Study design

TODAY (ClinicalTrials.gov: NCT05102396) is a pragmatic, randomized trial, conducted by academic investigators from multiple centers. The name is an acronym derived from the title “***TO*** *pical oxybutynin for hyperhi* ***D*** *rosis:* ***A*** *topic or s* ***Y*** *stemic effect*”.

**Figure 1:**
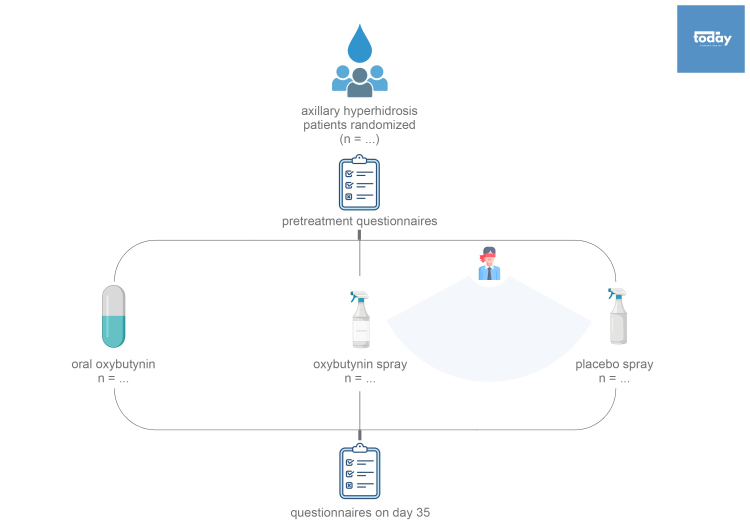
Study design of the TODAY clinical trial (topical oxybutynin for axillary hyperhidrosis).

The study design is Prospective Randomized Open Blinded Endpoint (PROBE) and approximately 21 patients on anticholinergic treatment with a confirmed diagnosis of HH will be recruited, as illustrated in Figure 1.

The TODAY trial is part of a register of all patients with HH admitted at two centers in Brazil. The first 20 eligible individuals among these patients diagnosed with axillary HH will be invited to take part in the trial, which will involve randomization to withdraw other HH treatments for 30 days. The baseline characteristics and the outcomes will be recorded using a Case Report Form (CRF).

The Brazilian Ministry of Health’s National Research Ethics Commission (CONEP - Comissão Nacional de Ética em Pesquisa) approved the study protocol (CAAE 30283520.0.0000.5479; decision 3.978.439). The study will follow the protocol and will be entirely compliant with Helsinki Declaration ethical principles and the International Council for Harmonisation Guideline for Good Clinical Practice. The study protocol requires informed consent from each patient before any study procedures are initiated.

Clinical diagnoses will be made by a trained team member using standard clinical assessments; imaging is unnecessary. Patients will be considered eligible if they have a symptomatic HH score ≥ 52 on the QoL scale, have signed the informed consent form (ICF), responded to the HDSS, are aged from 18 to 45 years, and have no history of sympathectomy. The authors have listed detailed inclusion and exclusion criteria in Table 1.

**Table 1:** Inclusion and exclusion criteria for the TODAY clinical trial (topical oxybutynin for axillary hyperhidrosis).

Inclusion criteria
1. Patients aged ≥ 18 years and ≤ 45 years
2. Patients who have not been treated with other medication or other treatment method therapeutic for the disease in the previous 30 days
3. Quality of life (QOL) ≥ 52
Exclusion criteria
1. Patients with hypersensitivity to oxybutynin hydrochloride
2. Patients who have been treated with other medication or other treatment method therapeutic for the disease in the previous 30 days
3. Patients with symptoms related to menopause
4. Patients with signs of skin changes in the axillary area
5. Pregnancy. Women with the potential to conceive and gestate who are not using contraceptive strategies and have not had a negative pregnancy test
6. Patients with Covid in the contagious phase (PCR+)

### Randomization and intervention

#### Randomization

Participants will be randomized into three treatment groups using a computer-generated randomization schedule prepared in advance of the study. In Group A, patients will be given oxybutynin at a dosage of 2.5 mg once a day, at night, for the first 7 days, followed by 2.5 mg twice a day from day 8 to day 21 and then 5 mg twice a day from day 22 to day 35. Group B will be given a topical placebo similar to the oxybutynin spray administered in two sprays into each armpit twice a day for 35 days. Group C will be given a topical oxybutynin 10% spray, also administered in two sprays into each armpit twice a day for 35 days.

**Figure 2:**
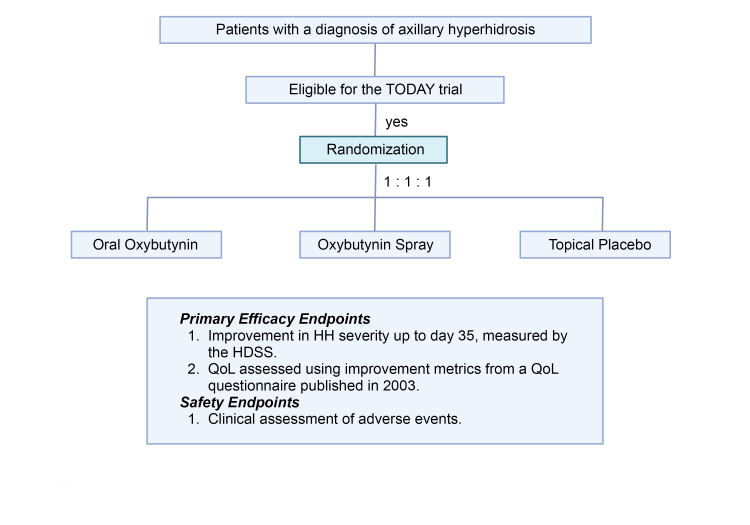
Flow diagram for the TODAY clinical trial (topical oxybutynin for axillary hyperhidrosis). QoL = quality of life; HH = hyperhidrosis; HDSS = Hyperhidrosis Disease Severity Scale.

Baseline characteristics will be recorded, and will be randomized electronically at a proportion of 1:1:1, balance with random sized blocks. This study will employ an open blinded endpoint design (Figure 2).

The REDCap validated software application will be used to generate a randomized list of variable sized blocks. In order to enroll a patient on the trial, the investigators will have to access the trial site and fill out a simple medical registration form.

#### Intervention

Patients will go through an initial screening at the first visit, followed by administration of two questionnaires: an HH-specific QoL form and the HDSS. Later, they will be randomized into one of three groups.

Both questionnaires, the QoL and the HDSS, will be administered at baseline and again on day 35, which is the last study visit.

### Quality of life questionnaire

To assess quality of life, each patient will answer a specific questionnaire^15^ . The maximum score is 100 points. If a question is not applicable to a patient’s situation or clinical condition, the authors will make proportional adjustments. The negative impact on QoL before treatment was classified into five satisfaction levels, calculated from the total score on the scale (varying from 20 to 100). A score greater than or equal to 84 indicates very bad QoL; scores from 68 to 83 indicate bad QoL; scores from 52 to 67 indicate good QoL; scores from 36 to 51 indicate very good QoL; and scores from 20 to 35 indicate excellent QoL. Improvements in QoL after treatment were also classified into five different levels. If the total score exceeded 84, QoL was considered much worse; from 68 to 83, slightly worse; from 52 to 67, unchanged; from 36 to 51, slightly better; and from 20 to 35, much better.

### Hyperhidrosis disease severity scale

Patients assessed the severity of their sweating using the HDSS. Developed by the International Hyperhidrosis Society®, the HDSS was designed to be a rapid assessment tool that employs just one question with four possible responses. The responses measure the impact that HH has on the patient’s daily life and their tolerance of the symptoms. The HDSS is simple, facilitating response and minimizing the likelihood of errors, thus optimizing the efficiency of medical assessments. This straightforward approach guarantees patients’ ability to rapidly and precisely convey the severity of their condition to healthcare providers. Varella et al.^16^ translated the HDSS into Portuguese, guaranteeing its applicability and utility in Portuguese-speaking populations. The translation maintains the scale’s integrity and efficacy, enabling consistent assessment of patients using different languages. The response options reflect the patient’s tolerance of diaphoresis symptoms and the extent of the negative impact on their daily life. Level 1 indicates unnoticeable sweating that never interferes with daily activities; level 2 is tolerable sweating that sometimes interferes with daily activities; level 3 is barely tolerable sweating that frequently interferes with daily activities; and level 4 is intolerable sweating that always interferes with daily activities. Patients should assign a specific score for each area affected by HH. For data analysis, the authors calculate the improvement delta from the HDSS scores: HDSS before treatment (week 0) minus HDSS after treatment (at the follow-up visit) gives the delta. A delta of 0 indicates no improvement; a delta of 1 or 2 indicates slight improvement; and a delta of 3 is considered a significant improvement.

### Primary efficacy endpoints

The endpoints for assessment of therapeutic efficacy will be improvement in HH severity up to day 35, measured by the HDSS [Time: day 35]. A treatment responder is defined as any participant whose initial HDSS score was 3 or 4 and who achieves an improvement of at least 1 point by day 35 and QoL will be assessed using improvement metrics from a quality of life questionnaire published in 2003.

### Safety endpoints

Clinical assessment of adverse events and severe adverse events will be recorded as the number of participants who undergo adverse events, including dry mouth and cutaneous symptoms.

## DISCUSSION

Hyperhidrosis is known as overactivity of sweat glands and is a common condition that predominantly affects young people during the maturation phase. The options for treatment of the condition are a subject of debate and axillary sprays containing oxybutynin have not been tested.

### Assessment of sweating

Devices that measure evaporation of sweat from the skin or the sweat can quantitatively assess diaphoresis. However, such instruments may not accurately reflect the daily discomfort undergone by individuals with HH. Therefore, patient-reported results are crucial to assess the impact of HH.^17^ The HDSS and the QoL questionnaire objectively assess the progression of HH. QoL assessments are essential tools in medical research, because they enable collection of high-quality data for diagnosis and monitoring of patients’ results. The impact of HH on QoL is well-documented, with easy-to-use questionnaires that are essential for clinical assessments.

### Impact on quality of life

The severity and location of HH can cause significant embarrassment, discomfort, and severe social, professional, and psychological problems, affecting daily activities and professional careers. Studies show that patients normally report bad or very bad QoL before treatment, because those whose QoL is better do not generally seek treatment. The main objective of HH treatment is to reduce production of sweat, which, when achieved, leads to improvements in QoL, relieving the psychological burden. These improvements are in spite of the potential side effects, generally considered insignificant by patients. In addition to impacting QoL, HH can also cause psychological and relational disorders, especially because it very often emerges during childhood and adolescence, which are critical periods for mental health development.

### Psychological and social aspects

Anxiety disorders affect from 12.2% to 48.6% of the general population and frequently cause reduced QoL. These are chronic, recurrent, and heterogeneous disorders that are often related to poor general health and negative results. Previous studies have shown that people with HH often have higher anxiety levels, demonstrating the importance of covering psychosocial factors in management of these patients. Studies indicate that treatment of HH with oral anticholinergics achieves significant improvement in anxiety symptoms, probably because sweating is controlled. Anxiety and HH may share common paths related to an overactive autonomic nervous system; a type of intermittent dysautonomia. Patients with HH suffer from both the discomfort of excessive sweating and from fear of negative social judgment, leading to avoidance, social isolation, or hypervigilance.

### Study expectations

We expect that the oxybutynin spray shows a trend to, at least, non-inferiority to oral oxybutynin and is superior to placebo in terms of efficacy.

### Study limitations and future directions

**Figure 3:**
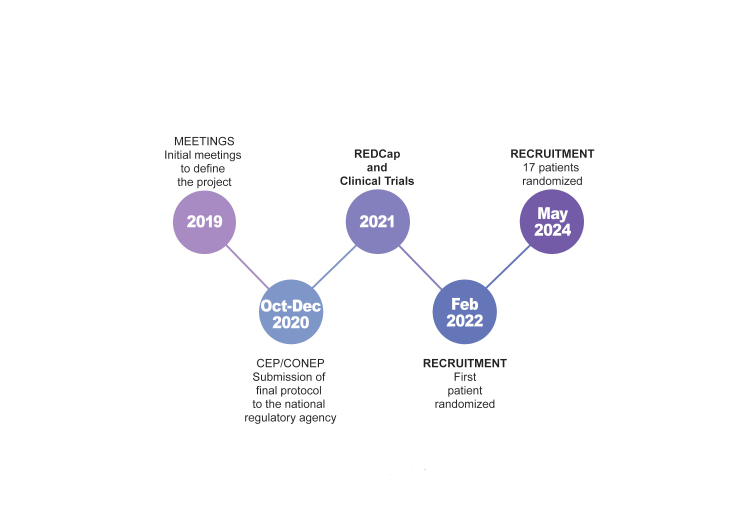
Timeline for the TODAY clinical trial (topical oxybutynin for axillary hyperhidrosis). CEP = Comitê de Ética em Pesquisa; CONEP = Comissão Nacional de Ética em Pesquisa.

This is the first study to investigate the impact of topical oxybutynin on QoL and HDSS scores. Limitations include the small sample size for a phase II trial and the impact of the COVID-19 pandemic, which restricted patient recruitment and access to HH diagnosis. After conclusion of the current phase II trial, future investigations will involve larger groups of patients treated with topical oxybutynin for prolonged periods and will include detection of oxybutynin in the blood using Liquid Chromatography-Mass Spectrometry (LC-MS), planned to understand the long-term results (Figure 3).

## CONCLUSIONS

The TODAY study is a phase II trial with a Prospective Randomized Open Blinded Endpoint (PROBE) design that will recruit patients diagnosed with axillary HH. The objective is to determine and assess the impact of topical oxybutynin on axillary HH. The study results will help guide medical decision-making with respect to the best way to manage axillary hyperhidrosis.
