# Supplementary material for: Topical oxybutynin deodorant for axillary hyperhidrosis: a topic or a systemic effect? Rationale and design of the phase II today trial
Source: J Vasc Bras. 2025 Feb 21;24:e20240098. doi: 10.1590/1677-5449.202400982 (PMC11864779; doi:10.1590/1677-5449.202400982)
Supplement: Translation

# Translation: Desodorante tópico de oxibutinina para hiperidrose axilar: efeito local ou sistêmico? Fundamentação e desenho do estudo de fase II today

**DOI:** 10.1590/1677-5449.202400981

**Authors:** Samantha Neves, Giuliano Giova Volpiani, Alexia Paganotti, Felipe Menegueti, Ricardo Sanchez Boix, Roberto Augusto Caffaro, Vanessa Prado dos Santos, Eduardo Ramacciotti

**Affiliations:** 1 Irmandade Santa Casa de Misericórdia de São Paulo, São Paulo, SP, Brasil.; 2 Universidade Federal da Bahia, Faculdade de Medicina da Bahia, Salvador, BA, Brasil.; 3 Science Valley Research Institute, São Paulo, SP, Brasil.; 4 Loyola University Medical Center, Hemostasis & Thrombosis Research Laboratories, Maywood, IL, EUA.

**Keywords:** hiperidrose, oxibutinina, anticolinérgico, hiperidrose axilar

## Abstract

Os anticolinérgicos melhoram a qualidade de vida com redução da sudorese em pacientes com hiperidrose, mas permanece incerto se a aplicação tópica exerce efeitos locais ou sistêmicos. O objetivo principal deste estudo é avaliar o impacto da oxibutinina tópica na hiperidrose axilar. Vinte pacientes com hiperidrose axilar serão randomizados em três grupos. O Grupo A receberá 2,5 mg de oxibutinina oral com diferentes posologias do primeiro ao 35º dia. O Grupo B receberá um placebo tópico na forma de *spray*, e o Grupo C receberá um *spray* de oxibutinina a 10%, com duas borrifadas em cada axila duas vezes ao dia por 35 dias (produto em investigação). O desfecho primário será a avaliação da eficácia do *spray* tópico de oxibutinina, avaliada pelo número de pacientes que apresentam melhora na gravidade da condição. O estudo TODAY mostrará evidências sobre os efeitos da oxibutinina tópica, avaliando seu impacto na hiperidrose axilar.

## INTRODUÇÃO

A hiperidrose (HH) é uma condição caracterizada por sudorese excessiva que vai além do necessário para regular a temperatura corporal^1^ . Afeta aproximadamente 15,3 milhões de indivíduos na América do Norte^2^ . A etiologia da HH primária permanece mal definida, mas se acredita que envolva hiperatividade do sistema nervoso simpático devido a predisposições genéticas^3^ . A HH tem um impacto significativo na qualidade de vida (QoL) e está associada a estigma social e profissional semelhante a condições crônicas como psoríase severa. Também há taxas mais altas de depressão e ansiedade relatadas entre indivíduos com HH^4^ . A HH primária, também conhecida como idiopática, responde por cerca de 90% dos casos de HH, sendo a axila a área mais comumente afetada^3^ . Essa forma de HH geralmente começa na adolescência e tende a piorar com o passar dos anos^5-7^ .

As opções de tratamento atuais para HH, incluindo simpatectomia toracoscópica assistida por vídeo (VATS), injeções de toxina botulínica, várias terapias tópicas e anticolinérgicos orais, exibem eficácia variável e podem causar efeitos colaterais significativos^8,9^ . Um estudo de longo prazo envolvendo 1.685 casos de HH primária tratados com oxibutinina oral mostrou melhorias na qualidade de vida e diminuição da gravidade da HH, medida pela Escala de gravidade da doença da hiperidrose (HDSS), embora 24,9% dos pacientes tenham experimentado xerostomia severa^10^ . Existem relatos limitados sobre anticolinérgicos tópicos, e o glicopirrolato tópico em forma de lenço e creme, bem como a oxibutinina em forma de gel, demonstraram ser eficazes^11-14^ .

## FUNDAMENTAÇÃO E OBJETIVOS DO ESTUDO

A avaliação do *spray* de oxibutinina tópico, que é mais fácil de usar, não foi testada anteriormente. O benefício de um *spray* de oxibutinina a 10% para HH axilar residiria em seus efeitos sistêmicos limitados, o que pode reduzir os efeitos colaterais simpatomiméticos observados com anticolinérgicos orais e, potencialmente, melhorar a adesão ao tratamento e melhorar tanto os escores de questionário de QoL^15^ quanto os escores de HDSS^16^ .

Este estudo de fase II visa a avaliar a eficácia de um *spray* tópico de oxibutinina a 10% aplicado duas vezes ao dia em comparação com placebo e oxibutinina oral em pacientes com HH axilar.

## MÉTODOS

### Desenho do estudo

O TODAY (ClinicalTrials.gov: NCT05102396) é um estudo pragmático, randomizado, conduzido por investigadores acadêmicos multicêntricos. Ele vem do acrônimo em inglês “***TO*** *pical oxybutynin for hyperhi* ***D*** *rosis:* ***A*** *topic or s* ***Y*** *stemic effect*”.

O estudo será aberto com avaliação de desfecho cega (PROBE, do acrônimo em inglês *Prospective Randomized Open Blinded Endpoint*) e incluirá aproximadamente 21 pacientes em terapia anticolinérgica com diagnóstico confirmado de HH, conforme ilustrado na Figura 1.

**Figura 1:** Desenho do estudo clínico TODAY (uso tópico de oxibutinina para hiperidrose axilar).

O estudo TODAY faz parte de um registro de todos os pacientes com HH admitidos em dois locais no Brasil. Desses pacientes, os primeiros 20 indivíduos elegíveis diagnosticados com HH axilar serão convidados a participar do ensaio, que envolve randomização para descontinuação de outros tratamentos para HH por 30 dias. As características basais e os desfechos serão registrados em um Formulário de Relato de Caso, o CRF (*Case Report Form*).

A Comissão Nacional de Ética em Pesquisa (CONEP) do Ministério da Saúde do Brasil aprovou o protocolo do estudo (CAAE 30283520.0.0000.5479; parecer 3.978.439). O estudo cumprirá o protocolo e aderirá totalmente aos princípios éticos da Declaração de Helsinki, especificações do Conselho Internacional de Harmonização e Boas Práticas Clínicas. O protocolo exige que cada paciente forneça consentimento informado antes de iniciar qualquer procedimento do estudo.

O diagnóstico clínico será realizado por um membro da equipe treinado utilizando avaliações clínicas padrão; a imagem não é necessária. Os pacientes serão considerados elegíveis se apresentarem escore de HH sintomático ≥ 52 na QoL, tiverem assinado o formulário de consentimento informado (ICF), preenchido a HDSS, tiverem idade entre 18 e 45 anos e não tiverem histórico de simpatectomia. Os autores detalharam os critérios de inclusão e exclusão na Tabela 1.

**Tabela 1:** Critérios de inclusão e exclusão do estudo clínico TODAY (uso tópico de oxibutinina para hiperidrose axilar).

Critérios de inclusão
1. Pacientes com idade ≥ 18 anos e ≤ 45 anos
2. Pacientes que não realizaram tratamento com outras medicações ou outro método terapêutico para a doença nos últimos 30 dias
3. Qualidade de vida (QOL) ≥ 52
Critérios de exclusão
1. Pacientes com hipersensibilidade a cloridrato de oxibutinina
2. Pacientes que realizaram tratamento com outras medicações ou outro método terapêutico para a doença nos últimos 30 dias
3. Pacientes com sintomas relativos à menopausa
4. Pacientes com sinais de lesões de pele na região axilar
5. Gravidez. Mulheres com potencial para concepção e gestação sem uso de estratégias contraceptivas e sem realização do teste de gravidez negativo
6. Pacientes com covid em fase de contágio (PCR+)

### Randomização e intervenção

#### Randomização

Os participantes serão randomizados em três grupos de tratamento usando um cronograma de randomização gerado por computador preparado antes do estudo. No Grupo A, os pacientes receberão oxibutinina na dosagem de 2,5 mg uma vez ao dia, à noite, durante os primeiros 7 dias, seguido de 2,5 mg duas vezes ao dia do dia 8º ao 21º e, então, 5 mg duas vezes ao dia do dia 22º ao 35º. O Grupo B receberá um placebo tópico como *spray* de oxibutinina aplicado com duas pulverizações em cada axila duas vezes ao dia por 35 dias. O Grupo C receberá um *spray* tópico de oxibutinina a 10%, também aplicado com duas pulverizações em cada axila duas vezes ao dia por 35 dias.

Serão registradas as características basais, e serão randomizados eletronicamente usando uma proporção 1:1:1, equilibrando com blocos de tamanhos aleatórios. Este estudo empregará um desenho de desfecho cego aberto (Figura 2).

**Figura 2:** Fluxograma do estudo clínico TODAY (uso tópico de oxibutinina para hiperidrose axilar). HH = hiperidrose; HDSS = Escala de gravidade da doença da hiperidrose; QoL = qualidade de vida.

O *software* REDCap validado gerará uma lista randomizada incorporando blocos de tamanhos variáveis. Para inscrever um paciente no ensaio, os investigadores devem acessar o *site* do ensaio e completar um formulário de registro médico simples.

#### Intervenção

Os pacientes passarão por uma avaliação inicial de triagem durante a primeira visita, seguida por dois questionários: um específico de QoL para HH e uma HDSS. Subsequentemente, eles serão randomizados em um dos três grupos.

Ambos os questionários, QoL e HDSS, serão administrados na linha de base e novamente no 35º dia, que é a última visita do estudo.

### Questionário de qualidade de vida

Para avaliar a qualidade de vida, cada paciente completará um questionário específico. Esse questionário.^15^ , consiste em uma pontuação máxima de 100 pontos. Se uma pergunta não se aplicar à realidade específica ou condição clínica do paciente, os autores farão ajustes proporcionais. O impacto negativo na QoL antes do tratamento foi classificado em cinco níveis de satisfação diferentes, calculados como a soma total da pontuação do protocolo (variando de 20 a 100). Uma pontuação igual ou maior que 84 indica uma QoL muito ruim; de 68 a 83 indica QoL ruim; de 52 a 67 indica QoL boa; de 36 a 51 indica QoL muito boa; e de 20 a 35 indica QoL excelente. A melhoria da QoL após o tratamento também foi classificada em cinco níveis de evolução diferentes. Quando o total foi superior a 84, a QoL foi considerada muito pior; de 68 a 83, ligeiramente pior; de 52 a 67, igual; de 36 a 51, ligeiramente melhor; e de 20 a 35, muito melhor.

### Escala de gravidade da doença de hiperidrose

Os pacientes usam a HDSS para avaliar a gravidade de sua sudorese. Desenvolvido pela International Hyperhidrosis Society®, a HDSS é projetada como uma ferramenta de avaliação rápida, apresentando uma única pergunta com quatro possíveis respostas. Essas respostas medem o impacto da HH na vida diária do paciente e a tolerância aos sintomas. A HDSS é simples, o que facilita a resposta e minimiza a probabilidade de erros, otimizando a eficiência das avaliações médicas. Essa abordagem direta garante que os pacientes possam transmitir rapidamente e com precisão a gravidade de sua condição aos provedores de saúde. Varella et al.^16^ traduziram a HDSS para o português, garantindo sua aplicabilidade e utilidade em populações de língua portuguesa. Essa tradução mantém a integridade e eficácia da escala, permitindo avaliações consistentes dos pacientes em diferentes idiomas. Essas opções refletem a tolerância do paciente aos sintomas de sudorese e a extensão do impacto negativo na vida diária. O grau 1 indica sudorese imperceptível ou sem interferência na vida diária; o grau 2, sudorese tolerável com interferência ocasional; o grau 3, sudorese moderadamente tolerável com interferência frequente; e o grau 4, sudorese intolerável com interferência constante. Os pacientes devem atribuir uma pontuação específica para cada local afetado pela HH. Para a análise de dados, os autores calculam um delta de melhoria a partir das pontuações da HDSS: HDSS antes do tratamento (semana 0) menos HDSS após o tratamento (na visita de acompanhamento) igual ao delta. Um delta de 0 indica nenhuma melhoria; um delta de 1 ou 2 indica ligeira melhoria; e um delta de 3 é considerado uma melhoria significativa.

### Resultados primários de eficácia

Os resultados para avaliação da eficácia terapêutica serão a melhoria na gravidade da HH até o dia 35, medida pela HDSS [Período: dia 35]. Um respondedor ao tratamento é definido como qualquer participante cujo escore inicial da HDSS foi 3 ou 4 e que alcançou pelo menos uma melhoria de 1 ponto na escala até o dia 35 e a avaliação da QoL usando métricas de melhoria de um questionário de qualidade de vida publicado em 2003.

### Resultados de segurança

A avaliação clínica de eventos adversos e eventos adversos graves foi registrada como o número de participantes que experimentaram eventos adversos, incluindo boca seca e lesões cutâneas.

## DISCUSSÃO

A HH, conhecida como hiperfunção das glândulas sudoríparas, é uma condição comum que afeta predominantemente indivíduos jovens durante sua fase de maturação. As opções de tratamento para essa condição são um tópico de debate, e os *sprays* axilares com oxibutinina não foram testados.

### Avaliação da sudorese

Dispositivos que medem a evaporação do suor da pele ou do suor podem avaliar quantitativamente a sudorese. No entanto, esses instrumentos podem não refletir com precisão o desconforto diário experimentado por indivíduos com HH. Assim, os resultados relatados pelos pacientes são cruciais para avaliar o impacto da HH^17^ . A HDSS e a QoL avaliam a progressão da HH objetivamente. As avaliações de QoL são ferramentas essenciais na pesquisa médica, pois permitem a coleta de dados de alta qualidade para o diagnóstico e monitoramento dos resultados dos pacientes. O impacto da HH na QoL é bem documentado, com questionários fáceis de usar e essenciais para as avaliações clínicas.

### Impacto na qualidade de vida

A gravidade e localização da HH podem levar a um embaraço significativo, desconforto e graves problemas sociais, profissionais e psicológicos, afetando as atividades diárias e as carreiras profissionais. Estudos mostraram que os pacientes normalmente relatam QoL ruim ou muito ruim antes do tratamento, pois aqueles com melhor QoL geralmente não procuram tratamento. O principal objetivo do tratamento da HH é reduzir a produção de suor, o que, quando alcançado, leva a uma melhora na QoL, aliviando os fardos psicológicos. Essa melhoria ocorre apesar dos potenciais efeitos colaterais, geralmente considerados insignificantes pelos pacientes. Além de impactar a QoL, a HH também pode causar distúrbios psicológicos e relacionais, especialmente porque, muitas vezes, aparece durante a infância e adolescência, um período crítico para o desenvolvimento da saúde mental.

### Aspectos psicológicos e sociais

Transtornos de ansiedade, que afetam de 12,2% a 48,6% da população geral, frequentemente levam a uma redução da QoL. São transtornos crônicos, recorrentes e heterogêneos, muitas vezes ligados à má saúde geral e a resultados negativos. Estudos anteriores mostraram que indivíduos com HH frequentemente experimentam níveis mais altos de ansiedade, destacando a importância de abordar os fatores psicossociais no tratamento desses pacientes. Pesquisas indicam que o tratamento da HH com anticolinérgicos orais leva a uma melhoria significativa nos sintomas de ansiedade, provavelmente devido à redução da sudorese. Ansiedade e HH podem compartilhar caminhos comuns relacionados ao sistema nervoso autônomo hiperativo, um tipo de disautonomia intermitente. Pacientes com HH experimentam tanto o desconforto da sudorese excessiva quanto o medo do julgamento social negativo, levando a evitação, isolamento social ou hipervigilância.

### Expectativas do estudo

Esperamos que o *spray* de oxibutinina mostre uma tendência de ser pelo menos não inferior à oxibutinina oral e superior ao placebo em termos de eficácia.

### Limitações do estudo e direções futuras

Este estudo é o primeiro a investigar o impacto da oxibutinina tópica nas pontuações de QoL e HDSS. As limitações incluem o pequeno tamanho da amostra para a fase II e o impacto da pandemia de covid-19, que restringiu o recrutamento de pacientes e o acesso ao diagnóstico de HH. Após a conclusão do atual estudo de fase II, pesquisas futuras envolverão grupos de pacientes maiores tratados com oxibutinina tópica por períodos prolongados e incluirão a detecção de oxibutinina no sangue usando LC-MS (*Liquid Chromatography-Mass Spectometry*, ou Cromatografia líquida acoplada à espectrometria de massa) planejado para entender os resultados a longo prazo (Figura 3).

**Figura 3:** Linha do tempo do estudo clínico TODAY (uso tópico de oxibutinina para hiperidrose axilar). CEP/CONEP = Comitês de Ética em Pesquisa/Comissão Nacional de Ética em Pesquisa.

## CONCLUSÃO

O estudo TODAY é um ensaio clínico de fase II, prospectivo, randomizado e com desfecho aberto-cego (PROBE) em pacientes diagnosticados com HH axilar. O objetivo é determinar e avaliar o impacto da oxibutinina tópica na HH axilar. Os resultados deste estudo ajudarão a orientar a tomada de decisões médicas em relação à melhor gestão da hiperidrose axilar.
